# A feasibility open-label clinical trial utilizing second-generation artificial intelligence based on the constrained-disorder principle in patients with Parkinson's disease

**DOI:** 10.1016/j.ibneur.2026.02.019

**Published:** 2026-02-26

**Authors:** Hillel Lehmann, Henny Azmanov, Yoav Hershkovitz, Noa Hurvitz, Samuel Agus, Marc Berg, David Arkadir, Yaron Ilan

**Affiliations:** a Departments of Medicine, Hadassah Medical Center, Faculty of Medicine, Hebrew University, Jerusalem, Israel; b Oberson Sciences Israel, Area 9, Denmark; c Stanford University, Palo Alto, CA, USA; d Departments of Neurology, Hadassah Medical Center, Faculty of Medicine, Hebrew University, Jerusalem, Israel

**Keywords:** Parkinson's disease, Levodopa, artificial intelligence, disorder, biological noise

## Abstract

**Background:**

Parkinson's disease (PD) is a neurodegenerative disorder treated with Levodopa, but long-term use often causes motor complications like "wearing-off," "on-off" effects, and Levodopa-induced dyskinesias, requiring careful management to balance benefits and risks. The Constrained Disorder Principle (CDP) defines biological systems by their inherent variability. CDP-based second-generation artificial intelligence (AI) systems introduce controlled variability into treatment regimens to counteract compensatory mechanisms that underlie drug loss of effectiveness.

**Objectives:**

This open-label, proof-of-concept feasibility clinical trial aimed to assess the technical feasibility and preliminary evidence of improved response to Levodopa by implementing algorithm-controlled therapeutic regimens.

**Methods:**

In this 14-week, open-label, single-center study, five PD patients used an app that randomized their Levodopa dosing times and dosages within pre-defined ranges. Primary outcomes were changes in the Unified Parkinson's Disease Rating Scale (UPDRS) and the Patient Global Impression of Improvement (PGI-I) scale. Statistical analysis was performed using the Wilcoxon signed-rank test with effect size calculations.

**Results:**

80% of patients demonstrated clinical improvement on the UPDRS, with a mean improvement of 4.4 points (p = 0.063, Cohen's d=0.82, 95% CI: −0.3–9.1), approaching but not exceeding the established minimal clinically significant difference threshold. Additionally, 60% reported a subjective improvement on the PGI-I scale. Furthermore, 80% of patients used the app daily, indicating high adherence.

**Conclusions:**

The results of this feasibility trial provide preliminary, hypothesis-generating evidence that CDP-based second-generation AI-driven personalized Levodopa dosing regimens may be technically feasible and potentially associated with clinical improvements in PD patients. However, the open-label design, small sample size, and absence of control conditions necessitate cautious interpretation. Adequately powered, randomized, double-masked controlled trials are needed to confirm these findings and rigorously evaluate efficacy and long-term effects.

## Introduction

Parkinson's disease (PD) is a common, chronic, degenerative nervous system condition. The pathological hallmark of PD is the presence of intraneuronal aggregates of alpha-synuclein (Lewy bodies). The movement disorder of PD arises mainly from the loss of dopaminergic neurons in the substantia nigra, leading to striatal dopamine depletion (Stoker et al., 2018). There is currently no cure for PD. Levodopa offers benefits in controlling motor symptoms but induces motor functions. Some patients may experience a "wearing-off" (worsening of symptoms) before the next dose is due. An "on-off" effect might also occur, with sudden, short periods of stiffness even when the next dose is not due. More than 50% of Parkinson's disease (PD) patients develop levodopa-induced dyskinesias (LID) within five years of treatment (Pahwa and Lyons, 2009).

Clinicians must balance the benefits of treatment against potential complications (Dragasevic-Miskovic et al., 2019). In many PD patients, identifying an effective dose timing that is not associated with unwanted side effects is challenging for chronic medication use (Straka et al., 2018).

Chronobiology determines the physiology of multiple metabolic and inflammatory pathways, which may be related to the pathogenesis of PD (Mukherjee and Maitra, 2015) (Haus, 2002). Circadian alterations are amongst the very first symptoms experienced in PD, and sleep alterations are present in most patients with overt clinical manifestations of PD (Mantovani et al., 2018). The biological clock has emerged as a potential therapeutic target in PD patients. Bright light, physical exercise, and melatonin are viewed as chronotherapeutic tools to alleviate motor disorders, sleep/wake alterations, anxiety, and depression in PD patients (Fifel, 2017, Fifel and Videnovic, 2019).

### Theoretical foundation: the constrained disorder principle

Inherent intra- and inter-patient variability patterns characterize most biological systems. This variability is reflected at the genetic, cellular, and whole-organ levels, including variability in heart rate, respiratory rate, and gait (Ilan, 2019a, Ilan, 2019b, Ilan, 2020a, Ilan, 2020b, El-Haj et al., 2019, Ilan, 2019a, Ilan, 2019b, Ilan-Ber and Ilan, 2019, Forkosh et al., 2020, Ilan, 2019c, Ilan, 2022a, Ilan, 2022b, Shabat et al., 2021, Ilan, 2023a, Rotnemer-Golinkin and Ilan, 2022, Finn and Misteli, 2019, Shields, 2009, Kox et al., 2011, Singh et al., 2018, Konig et al., 2016). A loss or change in physiologic variability is associated with poor prognosis (Nayyar et al., 2017, Avolio, 2013, Moon et al., 2016). The Constrained Disorder Principle (CDP) defines systems by their inherent variability. According to this principle, variability is essential for optimal functioning, provided it remains within dynamic boundaries, thereby enhancing the system's adaptation to internal and external perturbations (Ilan, 2022a).

In the context of the CDP, "disorder" refers specifically to controlled, bounded variability in biological parameters rather than stochastic noise, thermodynamic entropy, or random fluctuations. Unlike noise, which represents unwanted signal interference, CDP-based variability is adaptive and functional, constrained within dynamic physiological boundaries that optimize system performance. This framework is empirically grounded in neurobiological phenomena observed in PD, including: (1) the variable firing patterns of dopaminergic neurons in the substantia nigra, (2) the fluctuating motor response to levodopa in individual patients, and (3) the loss of normal motor variability (gait, tremor patterns) characteristic of PD progression (Ilan, 2020c, Bayatra et al., 2024, Hurvitz and Ilan, 2023).

The CDP-imposed constraints differ fundamentally from regularization methods in machine learning. While regularization techniques (L1, L2, dropout) penalize model complexity to prevent overfitting during training, CDP constraints define dynamic, patient-specific therapeutic boundaries within which controlled variability is introduced during treatment delivery. CDP operates at the intervention level rather than the model-training level, implementing real-time variability in medication dosing rather than constraining algorithmic parameters. The CDP framework emphasizes that biological systems function optimally not at fixed setpoints but within fluctuating ranges, and that therapeutic interventions should mirror this natural variability to prevent compensatory adaptation and maintain long-term efficacy (Niederer et al., 2019).

Standard dosing regimens often do not account for the natural variability in individual physiology, which can increase drug resistance (Ilan, 2020c, Bayatra et al., 2024, Hurvitz and Ilan, 2023). There is a growing emphasis on recognizing individual patient variability to personalize therapy. This approach uses individualized computational models and machine-learning tools to develop tailored strategies for guiding pharmacological treatment (Niederer et al., 2019). CDP-based second-generation artificial intelligence (AI) systems are designed to incorporate variability into therapeutic regimens to improve response and overcome drug tolerance (Ilan, 2022a, Hurvitz and Ilan, 2023, Ilan, 2020d, Ilan, 2021a, Ilan, 2022b, Sigawi and Ilan, 2023, Sigawi et al., 2023, Kessler et al., 2020, Ishay et al., 2021a, Kolben et al., 2021, Kenig et al., 2021, Azmanov et al., 2021, Potruch et al., 2020, Isahy and Ilan, 2021, Khoury and Ilan, 2019, Khoury and Ilan, 2021, Kenig and Ilan, 2019, Ilan, 2019d, Gelman et al., 2020, Ishay et al., 2021b, Ilan and Spigelman, 2020, Hurvitz et al., 2021, Ilan, 2021b, Gelman et al., 2022, Azmanov et al., 2022, Hurvitz et al., 2022, Kolben et al., 2023, Lehmann and Ilan, 2023, Adar et al., 2023, Ilan, 2023a, Sigawi et al., 2024a, Ilan, 2024a, Hurvitz et al., 2024, Ilan, 2024a, Y, 2024, Sigawi et al., 2024b).

This proof-of-concept study evaluated the feasibility of using a CDP-based second-generation AI system to enhance Levodopa response in patients with PD.

## Methods

### Study design

The clinical trial was an open-label, prospective, single-center, 14-week proof-of-concept feasibility study that examined the effect of an algorithm-based regimen on Levodopa response. Subjects were enrolled in a single-center trial at the Hadassah Medical Center in Jerusalem, Israel. The trial was registered with the Israeli Ministry of Health (MOH) on 2022–01–10 under registration number 010535.

As a proof-of-concept feasibility study, the sample size of 5 patients was determined based on resource constraints and the primary aim of assessing technical feasibility, patient adherence, and preliminary safety signals, while also generating preliminary effect size estimates for future sample size calculations. This sample size is consistent with Phase 0/I feasibility studies in digital health interventions.

The 14-week intervention period was selected to allow: (1) a 4-week run-in period for baseline app usage without dose modification, establishing baseline adherence patterns and familiarizing patients with the technology; and (2) a 10-week intervention period sufficient to observe changes in UPDRS scores, which have been shown to demonstrate meaningful changes over 8–12 week periods in pharmacological trials.

### Study population

Eligible subjects included those 18–75 years of age, males and females diagnosed with PD.

### Inclusion and exclusion criteria

Adult non-pregnant patients with a diagnosis of PD per UK Brain Bank criteria, Hoehn and Yahr stages 2–3, suffering from motor fluctuations for at least one and a half hours a day and treated with Levodopa at least three times a day, were enrolled. Patients were required to have a stable levodopa regimen for ≥ 3 months before enrollment and to have no planned medication changes during the study period.

Patients with evidence of severe infectious, malignant, autoimmune, or other systemic diseases were excluded from the study. Patients who could not provide written informed consent, lacked a smartphone, or could not adhere to the visit schedule and protocol were also excluded. Additional exclusion criteria included: cognitive impairment (Montreal Cognitive Assessment score <20), unstable psychiatric conditions requiring medication changes within 3 months, atypical Parkinsonism or secondary Parkinsonism, and participation in other interventional clinical trials.

### Second-Generation AI system: Technical specifications and algorithmic architecture

Altus Care™ is a cellular phone-based application and product of Area9 Innovation ApS that allows easy digitization of treatment plans or research protocols and remote implementation. An algorithm approach that provides random dosing regimens of selected drugs was used in the present study (Oberon Sciences, Israel). The system operates as a rule-based randomization engine implementing the following algorithmic architecture:

**Input variables**

• Allowable time windows for each daily dose.

**Processing mechanism:** The algorithm employs a pseudorandom number generator (using cryptographically secure random functions) to select:

1. Administration time: Time point randomly selected within the specified time window for each dose.
2. Constraint enforcement: Algorithm ensures total daily dose does not exceed physician-specified maximum.

Output:

• Daily personalized medication schedule delivered via push notifications to the patient's mobile device.
• Medication reminders at algorithmically determined times.

#### System classification

The term "second-generation AI" distinguishes this system from first-generation static digital health tools (e.g., simple medication reminders, static dosing schedules) by emphasizing: (1) personalization based on individual patient parameters, (2) dynamic variability introduction rather than fixed regimens, (3) outcome-driven design focused on clinical endpoints, and (4) implementation of biological principles (CDP) rather than purely algorithmic optimization. The system does not employ neural networks or machine learning in the traditional sense; instead, it implements algorithmic randomization within clinician-defined therapeutic boundaries.

#### Validation level

This study employed Level 1 (open-loop) implementation: the algorithm generates variable dosing without feedback-based adaptation during the intervention period. Future implementations include Level 2 (incorporating patient-reported outcomes for adaptation) and Level 3 (incorporating biomarker-based personalized variability signatures).

**Fig. 1:**
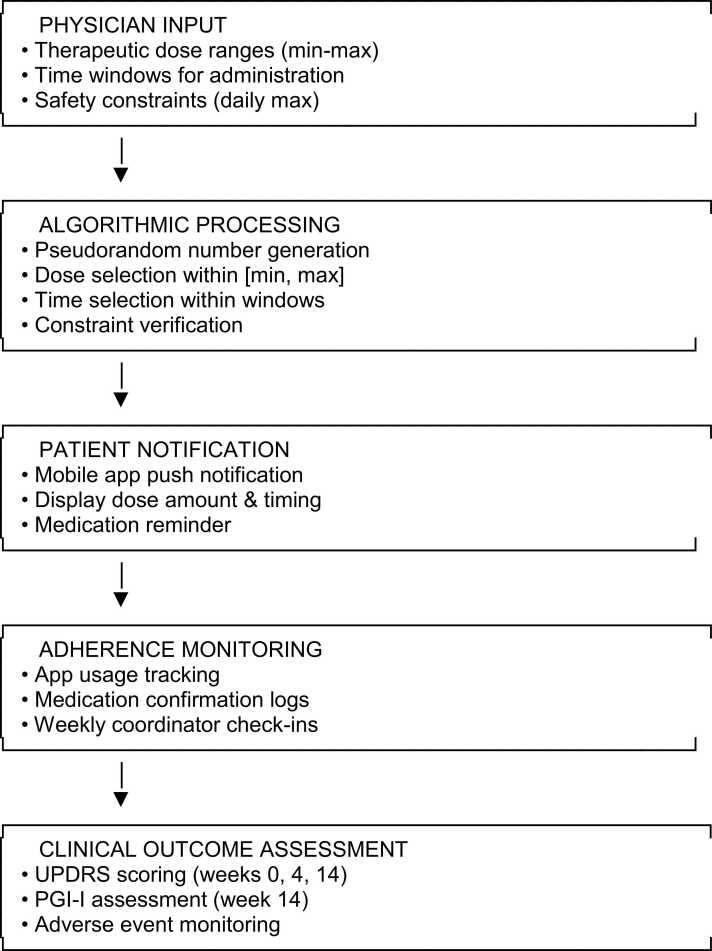
Algorithmic data flow schematic.

Fig. 1 provides a schematic representation of the data flow: Physician Input (therapeutic ranges) → Algorithmic Processing (randomization within constraints) → Patient Notification (mobile app) → Adherence Monitoring (app usage tracking) → Clinical Outcome Assessment.

The platform has been integrated with treatment algorithms that use second-generation AI to generate random adjustments to administration times within a pre-defined range, and it serves as a reminder for patients to take their medications. The app provides a personalized therapeutic regimen, creating variability in administration times within physicians' pre-defined administration time ranges.

### Study procedures

A convenience sample of eligible participants who fulfilled the inclusion and exclusion criteria was enrolled. Patients were recruited from the neurology clinic. Baseline clinical and laboratory parameters acquired at the screening included baseline vital signs, physical examination, and a neuromotor-clinical evaluation using the Unified Parkinson's Disease Rating Scale (UPDRS). The UPDRS was selected as the primary outcome measure as it represents the gold-standard, validated instrument for assessing PD severity across motor and non-motor domains. The UPDRS demonstrates established reliability (ICC 0.89–0.93), validity, and sensitivity to change in clinical trials. The PGI-I was included as a patient-reported outcome measure to capture subjective improvement, recognizing the importance of the patient perspective in assessing the clinical meaningfulness of interventions.

The Altus Care™ application was installed on the patient's cellular phone. During the first 4 weeks after enrollment, patients used the app without changing the dose or administration time. In coordination with the patient's treating physician, an individualized treatment plan was prepared for each patient within a pre-defined timing frame for its administration. Per protocol, the daily dose was set not to exceed the patients' dose before enrollment and was not administered more frequently than the patients' dose. No changes in concomitant PD medications were permitted during the study period. Patients maintained stable doses of dopamine agonists, MAO-B inhibitors, COMT inhibitors, and other adjunctive therapies throughout the intervention.

### Follow-up parameters

During the follow-up period, the research coordinator conducted weekly phone check-ins to assess the patient's clinical well-being and adherence to the treatment plan. At four weeks, the patients were evaluated by their treating neurologist, including baseline vital signs, physical examination, and a neuromotor-clinical evaluation using the Unified Parkinson's Disease Rating Scale (UPDRS). The same assessment was made at week 14 in addition to the Patient Global Impression of Improvement (PGI-I) questionnaire. All UPDRS assessments were conducted during "ON" states (within 1–2 h of levodopa administration) to minimize variability related to medication timing. Safety monitoring included adverse event reporting at each contact and systematic assessment of motor and non-motor complications at weeks 4 and 14.

### Statistics

The Wilcoxon signed-rank test for paired continuous nonparametric data was used for analysis. Effect sizes were calculated using Cohen's d for paired samples. 95% confidence intervals were calculated for mean changes. Given the exploratory nature of this feasibility study with two primary outcomes (UPDRS and PGI-I) and the small sample size, we did not apply corrections for multiple comparisons. This approach is consistent with feasibility trial methodology but increases the risk of Type I error, necessitating cautious interpretation. Descriptive statistics include medians and interquartile ranges for non-normally distributed variables.

## Results

### a. Subjects' demographics and safety measures

Out of 40 screened patients, 5 met the eligibility criteria and were recruited to participate in the study. Table 1 presents the demographics of the patients enrolled in the study. The participants, including three males and two females, had a mean age of 66. All patients completed the 14-week follow-up period and recorded no significant adverse events (Table 2).

**Table 1:** Patient characteristics.

**Characteristic**	**Value**
Age, mean (range), years	66 (56−72)
Male, number (%)	3 (60)
Time since diagnosis, median (range), years	5 (3−21)
Hoehn and Yahr stage, median (range)	2.5 (2−3)
Daily dosage of Levodopa, mean (range), mg	656 (375–1000)
Levodopa formulation, n (%)	
- Standard carbidopa/levodopa	4 (80)
- Extended-release formulation	1 (20)
Dosing frequency, mean (range), times/day	3.6 (3−5)
Concomitant PD medications, n (%)	
- Dopamine agonist	2 (40)
- MAO-B inhibitor	3 (60)
- COMT inhibitor	1 (20)
- Amantadine	1 (20)
Motor fluctuations duration, mean (range), hrs/day	2.4 (1.5–4.0)
[Baseline UPDRS total, mean (range)	42.6 (35−54)
Baseline UPDRS Part III (motor), mean (range)	28.4 (22−38)
Comorbidities	
- Ischemic heart disease, n (%)	1 (20)
- Type 2 diabetes mellitus, n (%)	1 (20)
- Hypertension, n (%)	2 (40)
- Hyperlipidemia, n (%)	1 (20)

**Table 2:** Algorithmic parameters by patient.

**Patient**	**Doses/Day**	**Time Windows**
1	4	7–9 AM, 12–2 PM, 5–7 PM, 9–11 PM
2	3	8–10 AM, 1–3 PM, 6–8 PM
3	5	7–9 AM, 11–1 PM, 3–5 PM, 7–9 PM, 10–12 AM
4	3	7–9 AM, 12–2 PM, 5–7 PM
5	4	8–10 AM, 12–2 PM, 4–6 PM, 8–10 PM

### b. Algorithm-guided variable dosing demonstrated preliminary evidence of clinical improvement in PD patients

**Fig. 2:**
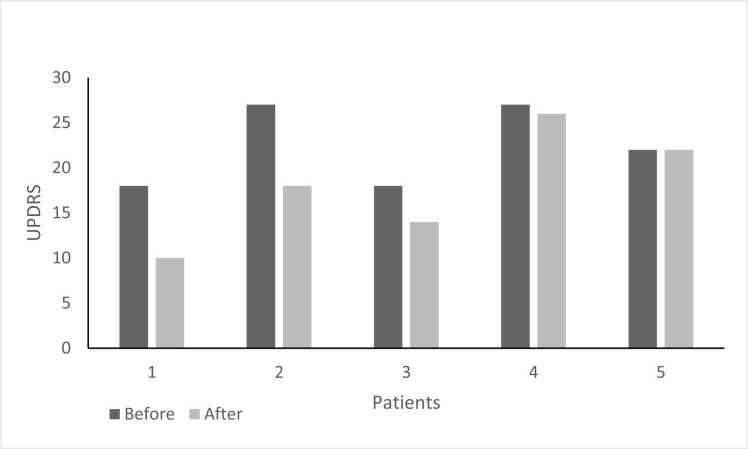

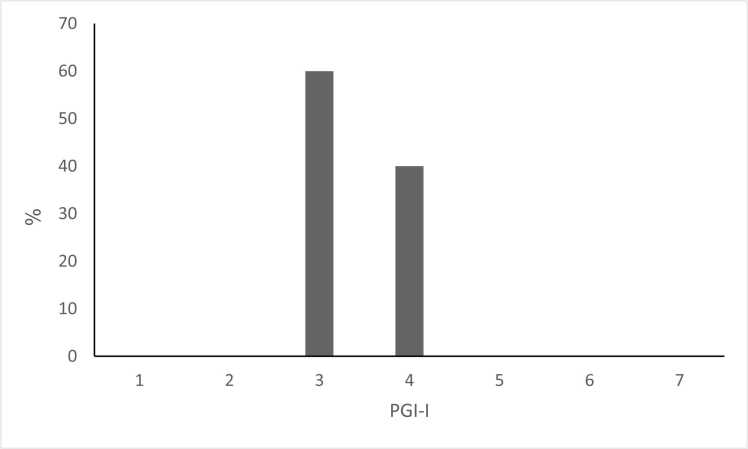

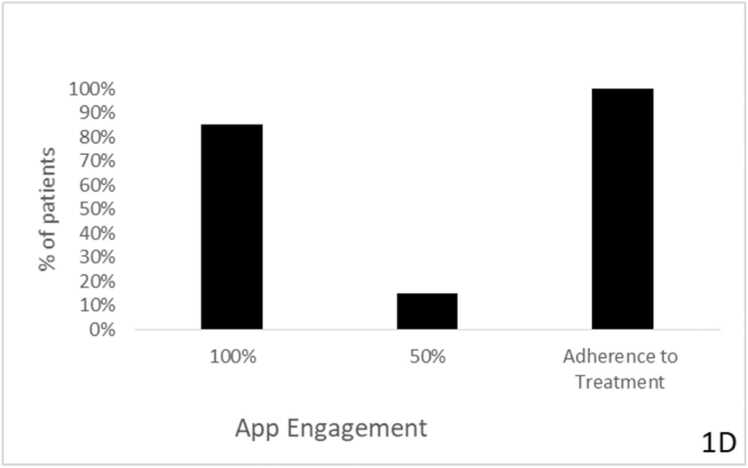
**A:** Effect of intervention on UPDRS score. The UPDRS score was evaluated in all patients before and after the intervention. Mean baseline UPDRS: 42.6 ± 7.2; Mean post-intervention UPDRS: 38.2 ± 6.8; Mean change: −4.4 points (95% CI: −0.3–9.1, p = 0.063, Cohen's d=0.82); Patients improving: 4/5 (80%); Patients unchanged: 1/5 (20%); Patients worsening: 0/5 (0%). **B:** patients' subjective improvement as assessed by the PGI-I questionnaire. (1 - significant improvement, 4 – no change, 7 – much worse). *Patient Global Impression of Improvement questionnaire (1 = considerable improvement, 4 = no change, 7 = much worse).* PGI-I score 3 (minimally improved): 3 patients (60%); PGI-I score 4 (no change): 2 patients (40%); No patients reported worsening (scores 5–7). **C:** Percentage of patients who adhere to treatment regimens and percentage of patients who use the app daily. Daily app usage: 4/5 patients (80%); Treatment adherence per phone questionnaires: 5/5 patients (100%); Mean daily app engagement time: 2.3 ± 0.8 min.

The CDP-based second-generation, AI-based personalized regimen was associated with preliminary improvements in Levodopa response. The UPDRS score improved in 4 patients (80%; p = 0.063), with a mean improvement of 4.4 points (95% CI: −0.3–9.1; Cohen's d=0.82), and did not change in 1 patient (20%); no patients had a worsening of their UPDRS score (Fig. 2A).

The mean improvement of 4.4 points approaches but does not definitively exceed the established minimal clinically significant difference (MCID) threshold of 5 points for UPDRS total score, suggesting preliminary clinical relevance that requires confirmation in larger controlled trials. Individual patient improvements ranged from 0 to 9 points, with 2 patients (40%) exceeding the MCID threshold. The Wilcoxon signed-rank test yielded p = 0.063, which does not achieve conventional statistical significance (α=0.05), reflecting the limited sample size and exploratory nature of this feasibility study.

For clinical context, placebo response rates in PD trials typically range from 16% to 50% for motor symptom improvement, with mean UPDRS improvements of 3–7 points commonly observed. The 80% response rate observed in this study exceeds typical placebo rates. However, the absence of a control group precludes definitive attribution to the intervention rather than placebo effects, expectancy effects, or regression to the mean.

Regarding the patient's subjective evaluation, three patients (60%) reported an improvement in their condition on the PGI-I scale (PGI-I = 3). In comparison, two patients (40%) reported no change in their condition (PGI-I = 4) (Fig. 2B).

First-generation digital health systems had low engagement rates among patients and physicians, while the CDP-based, second-generation, outcome-based system showed significantly higher engagement rates. Four patients (80%) used the app daily, and according to the study-coordinated phone questionnaires, all patients adhered to the treatment regimens (Fig. 2C).

## Discussion

This proof-of-concept feasibility study provides preliminary, hypothesis-generating evidence that CDP-based algorithmic modification of levodopa dosing may be technically feasible and potentially associated with clinical improvements in PD patients. However, the open-label design, small sample size, and absence of control conditions fundamentally limit causal inference. The findings must be interpreted with substantial caution and serve primarily to justify future rigorous investigation through adequately powered randomized controlled trials.

### Study limitations and validity considerations

The findings of this study must be interpreted with substantial caution, given significant methodological limitations that constrain the scope of permissible inference:

#### Open-label design

The absence of blinding introduces multiple threats to internal validity. Observer bias may have influenced UPDRS scoring, as raters were aware that patients were receiving an intervention expected to produce benefit. Patient expectancy effects may have influenced both objective performance and subjective reports, as patients knew they were receiving a novel technological intervention. The lack of a control group prevents differentiation between specific treatment effects and non-specific factors, including placebo response (which can be substantial in PD trials), regression to the mean, natural disease fluctuation, and the therapeutic alliance formed through frequent clinical contact.

#### Small sample size

With only 5 patients, the study lacks statistical power to detect treatment effects or generalize findings reliably. The observed p-value of 0.063 for the UPDRS change is suggestive but does not reach conventional statistical significance. Wide confidence intervals (from −0.3–9.1) reflect substantial uncertainty in the effect estimates. Individual patient variability in disease severity, levodopa responsiveness, and disease progression complicates interpretation.

#### Short follow-up

The 14-week intervention period is insufficient to assess long-term efficacy, safety, or durability of response. PD is a progressive neurodegenerative disease; longer follow-up is needed to determine whether observed improvements persist or whether compensatory mechanisms eventually overcome the CDP-based intervention.

#### Potential confounders

Natural fluctuations in PD symptoms, seasonal variations, lifestyle changes, and psychosocial factors associated with study participation may have contributed to the observed improvements, independent of the algorithmic intervention.

#### Single-center design

Recruitment from a single neurology clinic limits generalizability to broader PD populations with different demographic characteristics, disease severity, levodopa regimens, and healthcare contexts.

### Primary findings and mechanistic considerations

The data showed that 80% of subjects demonstrated improvements in UPDRS scores within 14 weeks of intervention, and subjective improvement was noted in 60% of patients. While these preliminary findings are encouraging, they require validation in controlled trials.

The CDP defines all complex biological systems in terms of inherent variability. It implies that the variability that characterizes systems is constrained by continuously changing dynamic boundaries (Ilan, 2023a, Ilan, 2022a, Sigawi et al., 2023, Ilan, 2023b). Loss of response to chronic medications or disease states reflects reduced variability or increased variability beyond borders (Ilan, 2023a, Ilan, 2022a, Sigawi et al., 2023, Ilan, 2023b). CDP-based second-generation AI systems implement variability signatures to improve response to chronic therapies and to overcome loss of response to chronic drugs (Ilan, 2023a, Ilan, 2023b, Ilan, 2024b). The system is designed to allow the treating physician to provide the app with a range of doses and administration times for the selected drugs. The algorithm randomly selects a number from the pre-specified range, ensuring it falls within the therapeutic window and the approved therapeutic regimen (Ilan, 2020c, Ilan, 2020d, Ilan, 2021a, Ilan, 2022b, Sigawi et al., 2024b, Ilan, 2024c, Ilan, 2025a).

The potential mechanisms by which CDP-based variability may enhance levodopa response in PD include: (1) prevention of compensatory downregulation of dopamine receptors through unpredictable dosing patterns, (2) optimization of synaptic dopamine availability by varying timing relative to circadian rhythms and meal timing, (3) reduction of tolerance development through variable drug exposure, and (4) improved alignment with natural physiological variability in motor control systems. However, these hypothetical mechanisms require direct experimental validation.

### Comparison with existing evidence

For comparison, typical levodopa dose optimization or adjustment strategies in clinical practice result in UPDRS improvements of 5–10 points, while medication switches or additions (e.g., adding dopamine agonists or MAO-B inhibitors) yield improvements of 8–15 points. The magnitude of improvement observed in this feasibility study (a mean of 4.4 points) is modest compared to that of these established interventions. It does not definitively exceed the MCID threshold, suggesting that any potential benefit of CDP-based dosing would likely be supplementary rather than transformative.

Perzon and Ilan (2025)A CDP-based second-generation AI system has shown preliminary evidence of improving response to therapeutic regimens in chronic diseases and potentially mitigating loss of response (Kessler et al., 2020, Ishay et al., 2021a, Kolben et al., 2021, Kenig et al., 2021, Azmanov et al., 2021, Potruch et al., 2020, Isahy and Ilan, 2021, Khoury and Ilan, 2019, Khoury and Ilan, 2021, Kenig and Ilan, 2019, Ilan, 2019d, Gelman et al., 2020, Ishay et al., 2021b, Ilan and Spigelman, 2020, Hurvitz et al., 2021, Ilan, 2021b, Gelman et al., 2022, Azmanov et al., 2022, Hurvitz et al., 2022, Kolben et al., 2023). In patients with heart failure who suffer from diuretic resistance, the system was associated with improved patients' clinical and laboratory parameters while reducing emergency room admissions and hospitalizations. It showed high engagement with the system among patients and caregivers (Gelman et al., 2023). Similarly, in patients suffering from chronic pain, introducing variability into therapeutic regimens improved the clinical response, overcoming the loss of response and enabling the use of a lower drug dose with fewer side effects (Sigawi et al., 2023). The system improved clinical response in multiple sclerosis patients, slowing disease progression (Sigawi et al., 2023). The system overcomes medication tolerance in patients with malignancy, improving clinical and radiological parameters in most subjects (Sigawi et al., 2024c). Similar beneficial effects were observed in patients with genetic diseases using the platform (Hurvitz et al., 2024). The inter- and intra-patient variability in response to Levodopa makes it challenging to achieve a sustained response (Pitz et al., 2020). The data from this feasibility study support the notion that the CDP-based second-generation AI system may provide a tool that continuously tailors the regimen to biological variability in a personalized way (Ilan, 2020d, Ilan, 2024c, Ilan, 2025a, Ilan, 2025b, Adar et al., 2025, Ilan, 2025c, Landau et al., 2025, Agmon et al., 2025).

In the present study, the first level of the algorithm, an open-loop system, was used. The second level personalizes the variability of the outcome using subjects' clinical and laboratory data. The third level is based on quantifying personalized signatures of variability in disease-relevant biomarkers and implementing them into the treatment regimens produced by the algorithm (Ilan, 2023a, Ilan, 2020c, Ilan, 2020d, Ilan, 2021a, Ilan, 2022b). In this way, each "evolution" of the AI-driven algorithm builds on the success of the previous version.

### Ethical considerations and regulatory framework

The deployment of algorithmic decision-making in clinical settings raises important ethical and regulatory considerations that were carefully addressed in this feasibility study:

#### Informed consent

All patients provided written informed consent after receiving detailed explanation of the study procedures, including: (1) the nature of algorithmic modification within physician-defined boundaries, (2) the investigational nature of the intervention, (3) potential risks and benefits, (4) the right to withdraw at any time without affecting their standard clinical care, and (5) continued access to their treating neurologist throughout the study. The consent process was conducted by a study physician using standardized consent documents approved by the institutional ethics committee, with opportunities for patients and families to ask questions.

#### Patient understanding of algorithmic decision-making

To ensure patient comprehension, the study team provided: (1) written materials explaining the CDP concept in lay language, emphasizing that medication amounts would vary within safe ranges, (2) visual demonstrations of the mobile application interface, (3) explanations that the algorithm would randomize dosing within boundaries pre-determined by their neurologist based on their individual therapeutic response, and (4) opportunities for questions before enrollment and throughout the study.

#### Algorithmic transparency

The algorithm's operation was made transparent to patients through: (1) explicit notification of each dose recommendation via the mobile application with specific dose amounts and timing, (2) explanation that recommendations remained within physician-approved ranges, (3) ability to view their personalized therapeutic ranges at any time in the app, and (4) option to contact the study team with questions or concerns at any time. However, the specific randomization sequence was not disclosed in advance to maintain study integrity and prevent anticipatory behavior that could confound results.

#### Regulatory compliance

This study was approved by the institutional ethics committee and registered with the Israeli Ministry of Health. The mobile application platform (Altus Care™) is certified as a Class II medical device in Europe. Future clinical deployment of CDP-based dosing systems would require: (1) regulatory approval as a medical device or software-as-a-medical-device (SaMD) with appropriate risk classification, (2) demonstrated safety and efficacy through randomized controlled trials meeting regulatory standards, (3) post-market surveillance mechanisms for ongoing safety monitoring, (4) clear labeling regarding intended use, contraindications, and limitations, and (5) healthcare provider training requirements.

#### Safety protocols

Patient safety was prioritized through multiple mechanisms: (1) all dose modifications remained strictly within each patient's established therapeutic window as determined by their treating neurologist based on their individual response history, (2) weekly phone contact with study coordinator to monitor for adverse events, medication side effects, or clinical deterioration, (3) scheduled in-person clinical assessments by neurologists at weeks 4 and 14, (4) patients retained ability to contact study team at any time with concerns, and (5) all treating physicians maintained final decision-making authority and could override algorithmic recommendations if clinically indicated.

### Future directions

These preliminary findings justify conducting an adequately powered, randomized, double-masked, controlled trial to evaluate efficacy rigorously. Such a trial should include an adequate sample size and use blinded assessment with independent raters blinded to treatment allocation to minimize observer bias. A control arm receiving standard fixed-schedule levodopa dosing with sham app notifications to control for attention and technology effects, and a longer follow-up of 6–12 months intervention period to assess durability of response and long-term safety.

In summary, utilizing CDP-based second-generation AI systems for patients with PD may potentially enhance their response to Levodopa. The findings from this feasibility study support the idea that implementing variability regimens warrants further investigation as a potential approach to improve reactions to chronic therapies. However, definitive conclusions about therapeutic efficacy require validation through adequately powered, randomized, controlled trials with appropriate blinding and more extended follow-up periods. This proof-of-concept study establishes feasibility and provides preliminary effect size estimates to guide future definitive trials.

## Authors' contributions

YI and DA conceptualized the study; HL, HA, YH, and NH conducted the research and analyzed the data. SA and MB analyzed the data. All authors contributed to the writing and review of the final version.

## CRediT authorship contribution statement

**Yaron Ilan:** Writing – review & editing, Writing – original draft, Conceptualization.

## Ethical Compliance

Approval for the study was received from the Hadassah Medical Center ethics committee.

## Patient consent

Written consent was received from each patient.

## Ethical Publication Guidelines

All authors have read and complied with the Journal's Ethical Publication Guidelines.

## Consent for Publication

Received from all authors.

## Funding

None.

## Conflicts of Interest

YI is the founder of Oberon Sciences, and SA serves as a consultant. MB serves as a consultant for Area9. No funding was received for the study.

## Data Availability

All data is available upon request.
